# Multi-nanolayered VO_2_/Sapphire Thin Film *via* Spinodal Decomposition

**DOI:** 10.1038/s41598-018-23412-4

**Published:** 2018-03-28

**Authors:** Guangyao Sun, Xun Cao, Yuanzheng Yue, Xiang Gao, Shiwei Long, Ning Li, Rong Li, Hongjie Luo, Ping Jin

**Affiliations:** 1 State Key Laboratory of High Performance Ceramics and Superfine Microstructure, Shanghai institute of Ceramics, Chinese Academy of Sciences, Dingxi 1295, Changning, Shanghai 200050 China; 2 University of Chinese Academy of Sciences, Beijing, 100049 China; 3 Section of Chemistry, Aalborg University, DK-9220 Aalborg, Denmark; 4 State Key Laboratory of Silicate Materials for Architectures, Wuhan University of Technology, Wuhan, 430070 China; 5 Thin Films and Nanostructures Group, Materials Science and Technology Division, Oak Ridge National Laboratory, 1 Bethel Valley Rd., Oak Ridge, Tennessee 37831 USA; 6 Department of Materials Science and Engineering, College of science, China University of Petroleum Beijing, No. 18 Fuxue RD, Beijing, 102249 China; 7 School of Materials Science and Engineering, Shanghai University, 99 Shangda Road, Shanghai, 200444 China; 8 Materials Research Institute for Sustainable Development, National Institute of Advanced Industrial Science and Technology, Nagoya, 463-8560 Japan

## Abstract

Coating of VO_2_-based thin film has been extensively studied for fabricating energy-saving smart windows. One of the most efficient ways for fabricating high performance films is to create multi-nanolayered structure. However, it has been highly challenge to make such layers in the VO_2_-based films using conventional methods. In this work, a facile two-step approach is established to fabricate multilayered VO_2_-TiO_2_ thin films. We first deposited the amorphous thin films upon sputtering, and then anneal them to transform the amorphous phase into alternating Ti- and V-rich multilayered nanostructure via a spinodal decomposition mechanism. In particular, we take advantage of different sapphire substrate planes (A-plane (11–20), R-plane (1–102), C-plane (0001), and M-plane (10-10)) to achieve different decomposition modes. The new approach has made it possible to tailoring the microstructure of the thin films for optimized performances by controlling the disorder-order transition in terms of both kinetic and thermodynamic aspects. The derived thin films exhibit superior optical modulation upon phase transition, significantly reduced transition temperature and hysteresis loop width, and high degradation resistance, these improvements indicate a high potential to be used for fabricating the next generation of energy saving smart windows.

## Introduction

There has been a long-standing demand for nanoscale phase separation owing to its connection to material functionalities. In recent years, intensive efforts have been devoted to understanding the connection^1–4^, and varied approaches for creating functional thin films have been reported, e.g., DNA-mediated self-assembly^5^, feedback-driven self-assembly^6^, and electrochemical techniques^7^. Conventional methods for fabricating thin films usually involve procedures such as template intermediates and/or post treatments, which make the fabrication process complicated and hence unstable. Self-assembly *via* spinodal decomposition is a promising solution since spinodal decomposition has proven to be efficient in controlling structural features at nanoscale^8–10^. The spinodal decomposition is a mechanism for the rapid decomposition of one thermodynamically stable mixture of liquids or solids into two coexisting phases^11^. In contrast to a nucleation-growth process that results in a random mixture of the two, spinodal decomposition is characterized by long-range spatial correlation, quasi-periodicity, and self-organization with a nearly sinusoidal composition modulation^12^. The structure with compositional fluctuations formed by spinodal decomposition tend to form at nanometer scale^13^. Thus, spinodal decomposition provides a practical route to produce a finely dispersed microstructure that can significantly enhance the material properties.

Most two-phase spinodal systems are consist of two phases with similar crystal structures and physical properties, e.g., metallic alloys^11^, SnO_2_-TiO_2_ system^14–16^, Al_2_O_3_-Cr_2_O_3_ system^17^ and AlN-SiC system^18^. This similarity limits the functionality of many decomposed systems. Spinodal decomposition of the TiO_2_/VO_2_ (TVO) system, which was discovered by Zanma and Ueda in 1998 and recently investigated in bulk materials by Z. Hiroi *et al*.^19,20^, is of great interest because of its unique properties of the two components (TiO_2_ and VO_2_). VO_2_ is a crucial component for achieving multi-functionalities of thin film since reversible first-order semiconductor-metal phase transition is accompanied by a drastic change in the optical, electrical, and magnetic properties between its two phases^21–24^. TiO_2_ is a commonly used wide band gap insulator and acts as an antireflection compound for VO_2_ to increase the transmittance of a thin film in both visible and infrared regions^25–27^. Therefore, transparent TVO thin films provide an attracting application in smart windows, that is, windows that capable of regulating solar/heat transmission for energy efficiency and comfort^28^. With the advantages of simple structure, automatic control without the use of switching devices, a new approach to enable large-scale producing TVO smart windows at a lower cost is highly desired.

Our previous work^29^ has shown the feasibility of spinodal decomposition on TVO thin films. However, the mechanism of spinodal decomposition in TVO system has not been fully uncovered, the decomposition-microstructure-property relation has not been explored. In the present work, TVO thin films with spinodal structures by performing room temperature sputtering-annealing are prepared using various sapphire substrates (A-plane, R-plane, C-plane and M-plane). We explore the decomposition-microstructure-property relation, reveal the mechanism of spinodal decomposition in TVO system, and we estimate the practical value of all the spinodal structures in smart window area. Our work provides an accurate self-assembled route to fabricate and control VO_2_-based multilayered thin films.

## Results and Discussion

Samples are abbreviated as A-A, A-R, A-C, A-M for amorphous TVO on A-plane (11–20) sapphire, R-plane (1–102) sapphire, C-plane (0001) sapphire, M-plane (10-10) sapphire substrates respectively. Representative spinodal decomposition samples on different sapphire substrates were abbreviated as SD-A, SD-R, SD-C, SD-M, and crystalline solid solution TVO on the previously mentioned sapphire substrates were abbreviated as C-A, C-R, C-C, C-M. Single component VO_2_ thin film samples were abbreviated as V-A, V-R, V-C, V-M and TiO_2_ thin film samples were T-A, T-R, T-C, T-M.

**Figure 1:**
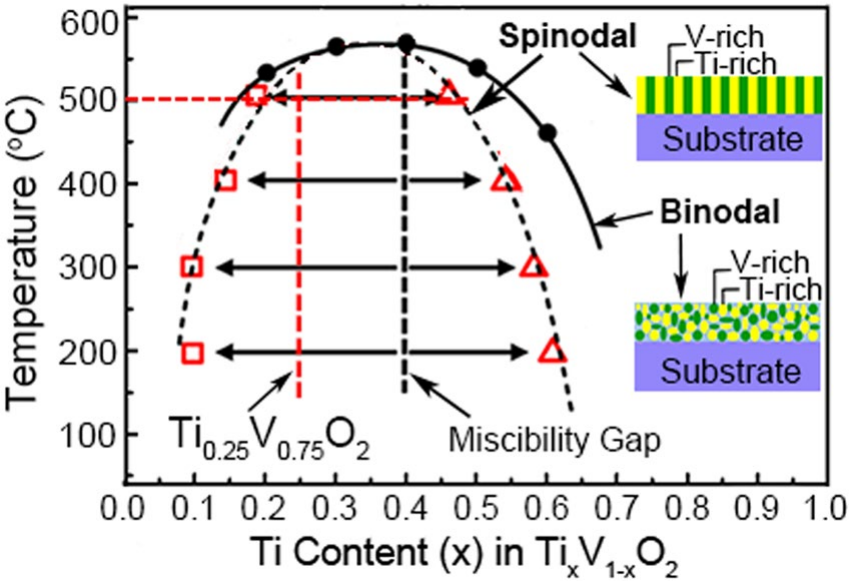
Phase diagram of the VO_2_-TiO_2_ system. Reprinted (adapted) with permission from (Z. Hiroi, *et al*. Spinodal Decomposition in the TiO_2_-VO_2_ System. *Chem. Mater*. **25**, 2202–2210 (2013). Copyright (2013) American Chemical Society.

Spinodal decomposition is illustrated on a phase diagram (Fig. 1). Usually, phase separation occurs whenever a material transforms into the thermodynamically unstable regions. The borderline of the unstable region to the stable region, often called the binodal curve, was first reported in TVO by Zamma and Ueda in 1998^19^ from calculations based on the common tangent construction of the free-energy diagram. Below the bimodal curve, there is a spinodal region, which was re-plotted from the literature data obtain from annealing experiments on the crystalline solid solution of Ti_0.4_V_0.6_O_2_ at four temperatures (200, 300, 400 and 500 °C)^19,30^. For compositions within the spinodal, a homogeneous solution is unstable against infinitesimal fluctuations in density or composition^31^.

It can be stated from Fig. 1 that, to reach the spinodal region, the material must cross the binodal region. Often phase separation occurs *via* nucleation and growth during the transition, and hence, spinodal decomposition cannot be observed. To avoid this scenario, a very fast transition by fast quenching is required to move the solid solution from the stable region to the spinodal region. This means spinodal decomposition needs rapidly quenching from high temperature (usually above 1000 °C) and post-annealing at the decomposition temperature. So far, this approach has been used in various spinodal systems^15,16,19,20^, and the combination of quenching (above 700 °C) with annealing (500 °C) method (Q-A) has been proven to be efficient in our TVO system (see Supplementary Fig. S1). With respect to our magnetron sputtering technique, the fast quenching from high temperature is not suitable since both the substrates and the thin films could easily fracture upon quenching. Therefore, to ensure the composition stability and uniformity of amorphous films, we first propose a two-step approach, namely, the room temperature sputtering-annealing (RTS-A) approach in our TVO system^29^, the schematic diagram of which is presented in Fig. S1. In our RTS-A route, spinodal decomposition is realized by directly annealing the amorphous TVO films, instead of high temperature and fast quenching.

Thin film composition was determined by XPS analysis (Fig. S2), the molar ratio of Ti to V is 0.33 for all the samples, and hence the composition can all be written as Ti_0.25_V_0.75_O_2_. The composition (Ti_0.25_V_0.75_O_2_) and the annealing temperature (500 °C) of the thin film are illustrated by the vertical and horizontal red dashed lines in Fig. 1, respectively. At the crosspoint, the final decomposed phases should be Ti_0.18_V_0.82_O_2_ (V-rich) phase and the Ti_0.49_V_0.51_O_2_ (Ti-rich) phase.

Researches^32–34^ have found that the epitaxial growth of VO_2_ and TiO_2_ on sapphire substrates has different out-of-plane orientations in terms of the sapphire type, as follows:

A-sapphire: (101) VO_2_(R)//(11–20) Al_2_O_3_, (101) TiO_2_(R)//(11–20) Al_2_O_3_;

R-sapphire: (101) VO_2_(R)//(1–102) Al_2_O_3_, (101) TiO_2_(R)//(1–102) Al_2_O_3_;

C-sapphire: (100) VO_2_(R)//(0001) Al_2_O_3_, (100) TiO_2_(R)//(0001) Al_2_O_3_;

M-sapphire: (001) VO_2_(R)//(10-10) Al_2_O_3_, (001) TiO_2_(R)//(10-10) Al_2_O_3_.

Confirmed by XRD results (Fig. S3), spinodal decomposition samples share the same orientations with the single component ones, thus we achieve different decomposition modes. Note that both the Ti-rich and V-rich peaks are located between the peak of pure VO_2_ and that of TiO_2_, the composition of the separated phases are V-doped TiO_2_ (Ti-rich phase) and Ti-doped VO_2_ (V-rich phase), respectively.

**Figure 2:**
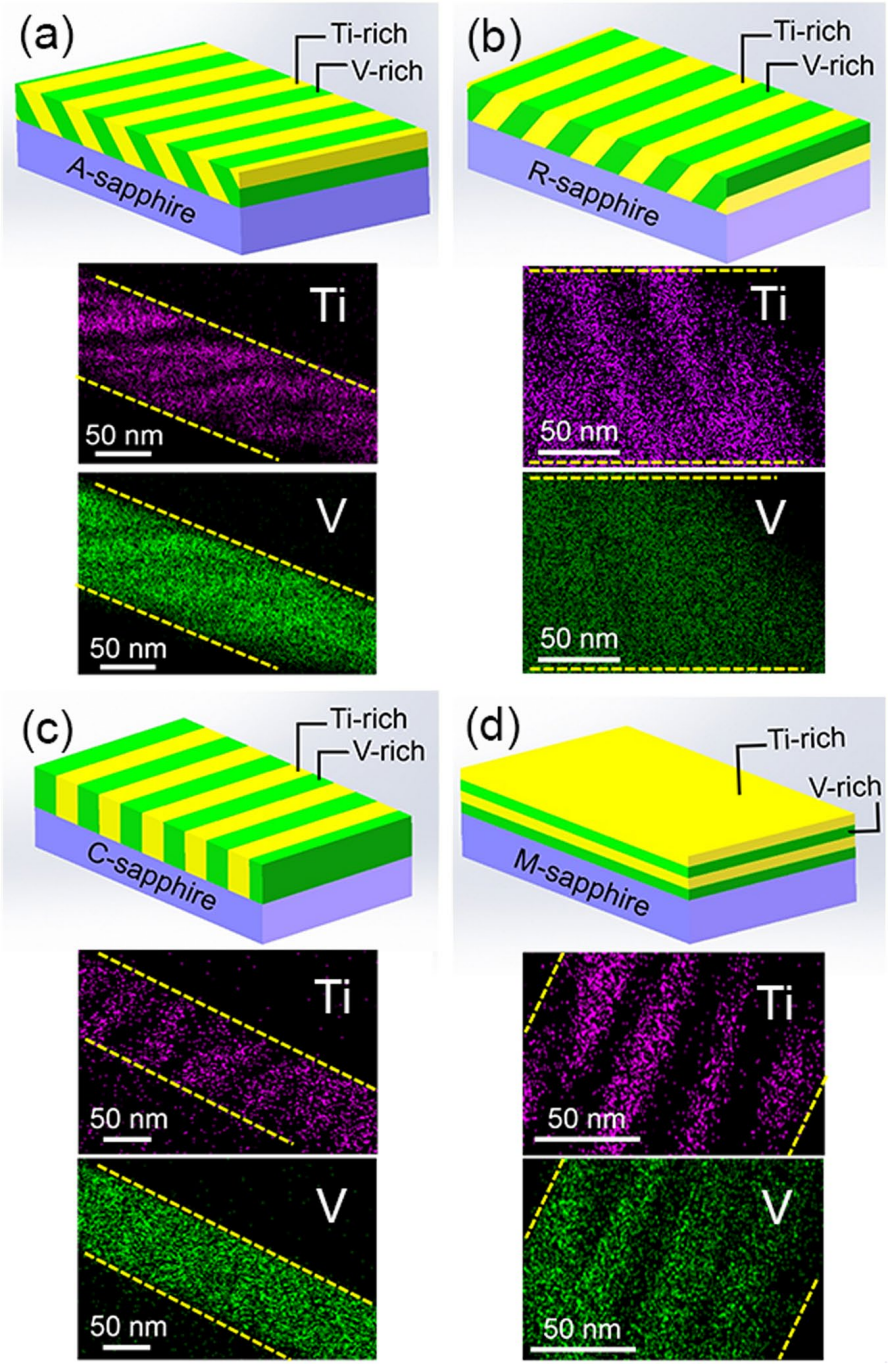
Schematic diagrams of the microstructure of spinodally decomposed TVO for (**a**) SD-A, (**b**) SD-R, (**c**) SD-C and (**d**) SD-M, and the related EDS elemental mapping (Ti and V) analyses of the selected area in Fig. S4.

Standard lattice parameters of rutile TiO_2_ are *a* = 4.582, *c* = 2.953 (PDF: 78-1510), and of tetragonal VO_2_ are *a* = 4.554, *c* = 2.8557 (PDF: 79-1655). The lattice mismatch in TVO system is 0.61% along the *a* axis and 3.3% along the *c* axis. In this case, spinodal decomposition modulation in the TVO system should occur along the *c* axis to minimize the elastic strain energy at the interface. The expected multilayers should be parallel to the (001) plane of TVO. This hypothesis is confirmed in TVO bulk crystals where spinodal decomposition occurs only for *hkl* reflections with *l* ≠ 0^20^. Similarly, spinodal decomposition occurs only along the *c* axis, i.e., [001] direction in the TiO_2_/SnO_2_ system^15,35^. Combined with the XRD results, we can infer the direction of decomposed multilayers will be slanted with respect to the substrate for SD-A and SD-R, perpendicular to the substrate for SD-C, and parallel to the substrate for SD-M. Schematic diagrams of the microstructure of the spinodally decomposed TVO thin films are shown in Fig. 2. Figure 2 also shows the cross-sectional EDS elemental mapping of Ti and V (originate from the STEM in Fig. S4), in high consistence with the schematic structures. The overall thickness of the TVO film was measured to be about 120 nm (Fig. S5), the thickness of Ti-rich layer is estimated to be ~20 nm(Fig. S4). Detailed high-resolution TEM analyses are shown in Fig. S5.

**Figure 3:**
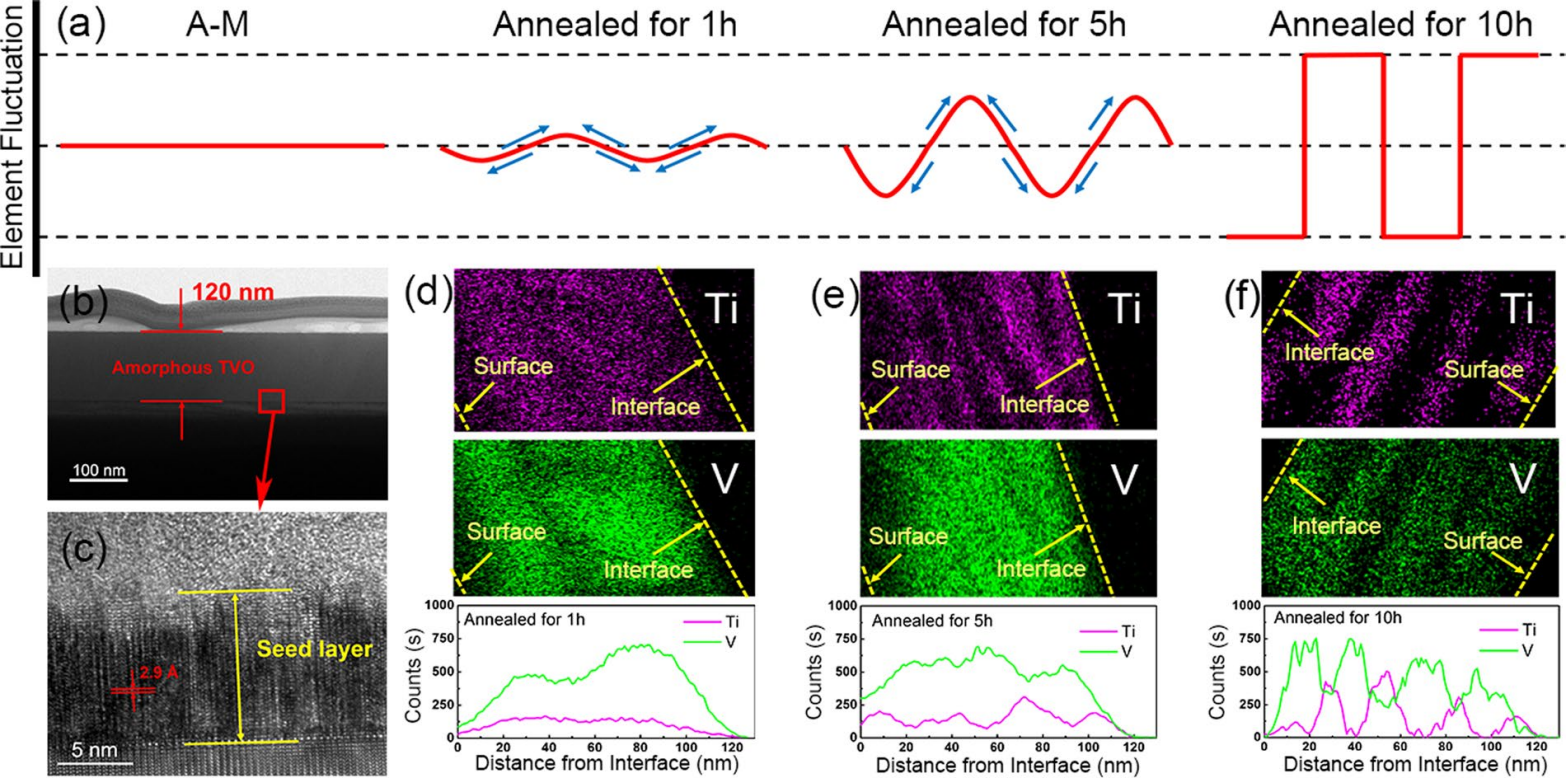
(**a**) Schematic diagram of element (Ti or V) content fluctuation in phase separation evolution progress. (**b**) TEM image of A-M sample. (**c**) High resolution TEM image of the select red square area in image (**b**). (**d**–**f**) EDS elemental mapping of the selected area (white square in Fig. S6) and line scanning of selected line (yellow line in Fig. S6) analyses for (**e**) sample annealing for 1 h, (**f**) sample annealing for 5 h, (**g**) sample annealing for 10 h. All the EDS line scanning images begin from the interface between TVO film and substrate and end at the surface of TVO film.

Evolution of the separated phases during spinodal decomposition was monitored on SD-M samples annealed for different annealing time, as shown in Fig. 3. Since there is no thermodynamic barrier to the reaction inside the spinodal region (Fig. 1), spinodal decomposition proceeds solely *via* diffusion mechanism, and the schematic diagram of the element (Ti or V) content fluctuation evolution is shown in Fig. 3(a). It is a sinusoidal wave curve, the amplitude increases along with annealing time and the amplitude is limited by the composition of original solid solution. The film thickness of amorphous sample (A-M) is about 120 nm, investigated by TEM (Fig. 3(b)) and the film surface is flat. TEM proves that A-M was predominantly amorphous since only few crystalline domains are present. Moreover, in HRTEM of Fig. 3(c), there is a pronounced epitaxial crystalline layer of ~10 nm forms in the interface between the substrate and TVO film. The interplanar crystal spacing is measured to be 2.9 Å, matching well with the 2*d* value calculated from XRD (002) peaks of sample C-M (64.45°, *d* = 1.46 Å) in Fig. S3(d). It is reasonable to state that the sapphire substrate is effective in promoting epitaxial growth and the epitaxial thin layer can act as seeds of the two separated phases in the following annealing process.

EDS elemental mapping, and EDS line scanning are shown in Fig. 3(d),(e) and (f) for annealing times of 1 h, 5 h, 10 h, respectively (originate from the STEM images in Fig. S6). The images of each EDS line scan, which starts from the interface between the substrate and TVO and ends at the surface of TVO film, agree well with the schematic diagrams in Fig. 3(a), supporting the diffusional scenario. At the beginning of annealing (1 h, Fig. 3(d)), the as-grown film is a homogeneous solid solution and only little fluctuation can be observed. With the annealing, fluctuations grow until individual phases can be identified (5 h, Fig. 3(e)), but the phase separation is incomplete and appears to be wavy lamellar structures at this stage. After sufficient annealing (over 10 h, Fig. 3(f)), the final equilibrium of phase separation with relatively sharp interface structure has been established. The final phase is stable with extending the annealing time at the fixed annealing temperature, because compared with the 10 h annealed sample, the longer time annealed (20 h) one displays similar XRD intensity (Fig. S7), similar transmittance spectra (Fig. S8(a and b)), and similar hysteresis loops (Fig. S8(c)).

**Figure 4:**
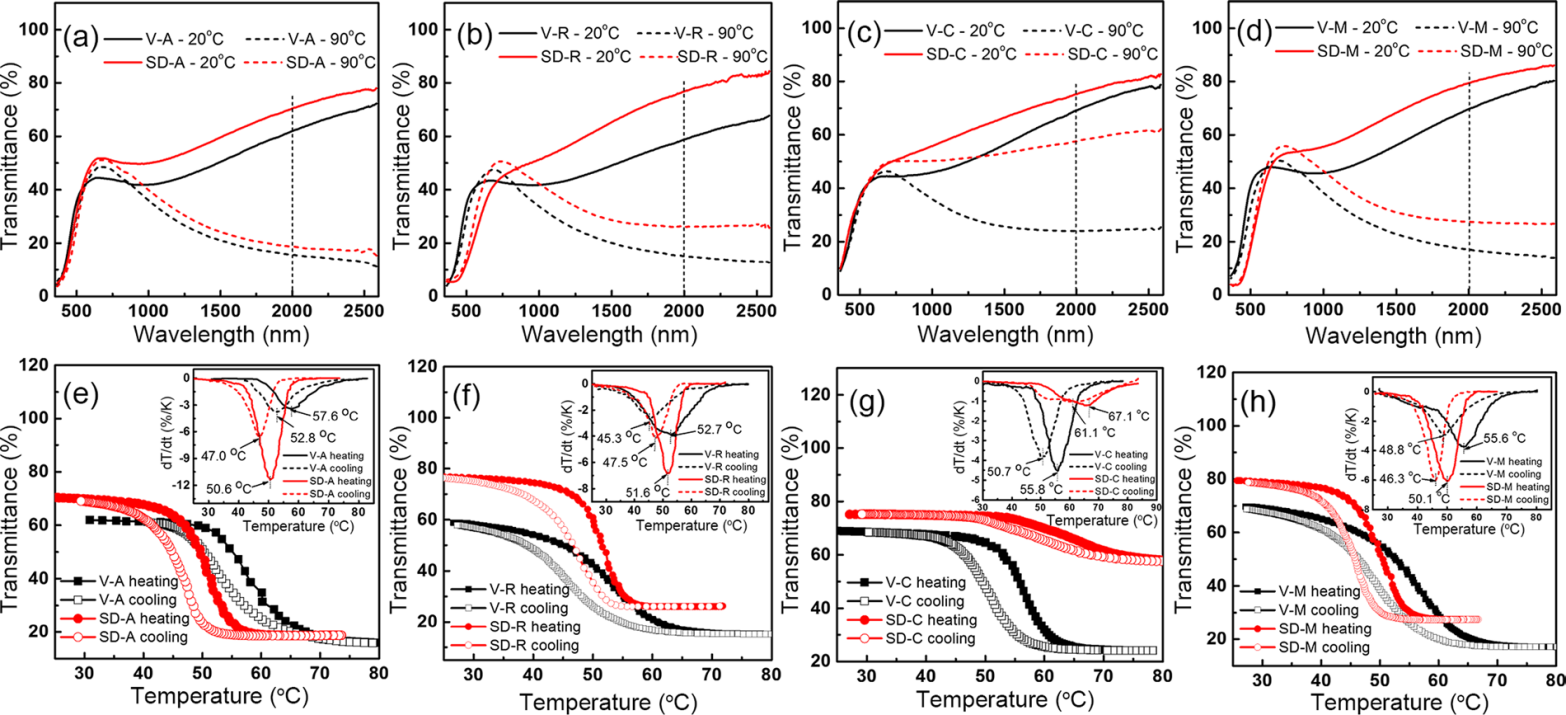
(**a**–**d**) Vis-near-infrared transmittance spectra at 20 °C and 90 °C of samples (**a**) V-A, SD-A, (**b**) V-R, SD-R, (**c**) V-C, SD-C, (**d**) V-M, SD-M. (**e**–**h**) emperature-varied transmittance hysteresis loops of samples (**e**) V-A, SD-A, (**f**) V-R, SD-R, (**g**) V-C, SD-C, (**h**) V-M, SD-M. Insets are the first-order differential curves of transmittance to temperature (dT/dt).

For application purposes, spinodally decomposed TVO thin films (SD-A, SD-R, SD-C, SD-M) are regarded as VO_2_ and TiO_2_ multilayer thin films. Based on the semiconductor-metal transition of VO_2_, those thin films are expected to be utilized as smart windows^27^. The vis-near-infrared transmittance spectra of the composite films were characterized at 20 °C (before phase transition) and 90 °C (after phase transition) to determine their optical modulation capability, as shown in Fig. 4(a–d). Transmittance spectra of single VO_2_ samples (V-A, V-R, V-C, V-M) are also shown for comparison. The application of VO_2_ for smart windows relies on the enhancement in both luminous transmittance (*T*_*lum*_) and solar modulating ability (*ΔT*_*sol*_), which are determined using the following equation^36^:

1 $${{\rm{T}}}_{{\rho }}=\int {\psi }_{{\rho }}({\lambda }){\rm{T}}({\lambda })d{\lambda }/\int {{\psi }}_{{\rho }}({\lambda })d{\lambda }$$

2 $${\rm{\Delta }}T={\rm{\Delta }}{T}_{sol,{20}^{^\circ }{\rm{C}}}-{\rm{\Delta }}{T}_{sol,{90}^{^\circ }{\rm{C}}}$$

where *T*(*λ*) is the transmittance at wavelength λ, *ρ* denotes *lum* or *sol* for calculations, *ψ*_*lum*_ is the standard efficiency function for photopic vision, and *ψ*_*sol*_ is the solar irradiance spectrum for an air mass of 1.5 (corresponding to the sun standing 37° above the horizon). The optical features of samples are summarized in Table 1.

**Table 1:** Optical properties of single VO_2_ samples and spinodal decomposition samples.

Sample	*T*_*lum*_ (%)		*T*_*sol*_ (%)		*ΔT*_*sol*_ (%)	*ΔT*_*2000nm*_ (%)	*T*_*c-heating*_ (°C)	*T*_*c-cooling*_ (°C)	*ΔT*_*c*_ (°C)
	20 °C	90 °C	20 °C	90 °C					
V-A	40.7	40.0	40.5	34.4	6.1	46.4	57.6	52.8	4.8
SD-A	42.7	39.7	45.4	35.8	9.6	51.7	50.6	47.0	3.6
V-R	39.8	38.7	39.6	33.0	6.6	43.7	52.7	45.3	7.4
SD-R	22.7	29.1	38.8	34.1	4.7	50.5	51.6	47.5	4.1
V-C	41.1	40.7	43.3	35.8	7.5	45.0	55.8	50.7	5.1
SD-C	41.5	42.1	48.6	45.3	3.3	17.6	67.1	61.1	6.0
V-M	45.0	41.8	44.5	36.5	8.0	52.5	55.6	48.8	6.8
SD-M	32.3	35.9	44.7	38.1	6.6	51.9	50.1	46.3	3.8

In Fig. 4(a–d) and Table 1, each spinodally decomposed TVO film (SD-A, SD-R, SD-C, SD-M) displays thermochromic properties, compare with the optical properties of the recently reported VO_2_-based thermochromic films prepared by magnetron sputtering (listed in Table S2), one can see that all the film can potentially be applied as smart coating material. However, compared to the single VO_2_, the relative tendency is not uniform across the four types. Comparing SD-A to V-A, the *T*_*lum*_ remains constant, while the *ΔT*_*sol*_ increases significantly from 6.1% for V-A to 9.6% for SD-A. The transmittance difference of typical wavelength of 2000 nm (*ΔT*_*2000* *nm*_) is measured as 46.4% for V-A and 51.7% for SD-A. For SD-R relative to V-R, and both *T*_*lum*_ and *ΔT*_*sol*_ decrease while *ΔT*_*2000*_ _*nm*_ increases. This indicates that the Ti-rich coatings slightly increase the total transmittance, but lead to a red shift of the absorption edge in Fig. 4(b), consistent with the function of TiO_2_ coatings in VO_2_ based thin films^37^. SD-C exhibits an increase in *T*_*lum*_, but more obvious decrease in *ΔT*_*sol*_ compared to V-C (Fig. 4(c)). The change is reasonable because the perpendicular orientation of the microscopic structure causes incomplete coverage of the V-rich layers on the substrate thus increases the total transmittance for the higher transparent property of Ti-rich layer^29^, and lowers the thermochromic functional area. SD-M displays relatively high thermochromic performance with *T*_*lum*_ of 32.3% at 20 °C, 35.9% at 90 °C, *ΔT*_*sol*_ of 6.6%, and *ΔT*_*2000*_ _*nm*_ of 51.9%. These data are slightly lower than those of V-M. For the relatively high-level Ti-doping in V-rich layers, the total transmittance improvement and red shift phenomena are similar to SD-R and SD-A. More detailed band gap calculation data are discussed and shown in Fig. S9.

As shown in Fig. 4(e–h), the hysteresis loops were measured at 2000 nm. Phase-transition temperatures of heating lines (*T*_*c-heating*_) and cooling lines (*T*_*c-cooling*_) are obtained from the peak value of the first-order differential curves (dT/dt), respectively^38^. Usually, *T*_*c-heating*_ is often used to represent the phase-transition temperature (*T*_*c*_). The hysteresis loop width (*ΔT*_*c*_) is estimated through *ΔT*_*c*_ = *T*_*c-heating*_-*T*_*c-cooling*_ (see the insets of Fig. 4(e–h) and Table 1**)**.

The *T*_*c*_ of the VO_2_ single crystal should be ~68 °C^39^, but our single VO_2_ samples are decreased to: 57.6 °C for V-A, 52.7 °C for V-R, 55.8 °C for V-C, and 55.6 °C for V-M. The decrease in *T*_c_ is ascribed mainly to the stress arising from ion bombardment during the deposition process and the mismatch in coefficient of thermal expansion between VO_2_ film and sapphire. For spinodally decomposed TVO films, only SD-C displays an increase in *T*_*c*_, others show decrease in *T*_*c*_. Jin *et al*. have indicated that the *T*_*c*_ reduction of VO_2_ film results mainly from doping and/or lattice stress^37^. Researches have found that the *T*_c_ value of VO_2_ would slightly increase by Ti doping^40,41^, which seems not to be the main influence factor of the variation. Generally, for lattice stress in VO_2_-based thermochromic multilayer film, the anisotropic compression pressure along the *c* axis of VO_2_ layer is the most effective strain, compressive stress along *c* axis causes the incerease in *T*_*c*_, while the tensile stress decreases *T*_*c*_. As we discussed above, spinodal decomposition in our TVO system occurs along the *c* axis to minimize the elastic strain energy. Since the *a* axis of TiO_2_ (*a* = 4.582, PDF: 78-1510) is larger than VO_2_, (*a* = 4.554, PDF: 79-1655), the *a* axis of V-rich layer should be under tensile stress while the *c* axis is under compressive stress in the V-rich phase, causing the reduction in *T*_c_ in SD-A, SD-R, and SD-M samples. For the V-rich layer in SD-C, the same influence from Ti-rich also exist. Nevertheless, since the orientation relationship between V-rich layer and the substrate is (100) VO_2_(R)//(0001) Al_2_O_3_, and the spinodally decomposed layers are perpendicular to the substrate, *b* axis and *c* axis of VO_2_(R) (tetragonal, *a* = 4.55, *c* = 2.86) are extended for the larger lattice paremeter of Al_2_O_3_(hexagonal, a = 4.75), schematic illustration of heteroepitaxial relationship and tensile stress in VO_2_ of VO_2_/Al_2_O_3_ interface could be found in our previous work^34^. Put these two competitive factors together, the *c* axis of V-rich layer is suffered tensile stress in SD-C sample at length.

Ladd has reported that anisotropic compression along *c* axis could theoretically reduce *T*_*c*_ by −12 °C/GPa^42^. From Table 1, the reduction of SD-A is 7.0 °C, SD-R is 1.1 °C, and SD-M is 5.5 °C, thus the anisotropic pressure is estimated as ≥0.58 GPa in SD-A, ≥0.1 GPa in SD-R, and ≥0.46 GPa in SD-M. Thus, we can state that spinodal decomposition is a new route to achieve huge anisotropic stress. Internal stress is of importance for high-frequency response VO_2_ applications, and this is confirmed by the reduction in hysteresis loop width (*ΔT*_*c*_) of SD-A, SD-R, and SD-M (Table 1).

Moreover, to evaluate the stability of spinodally decomposed films, durability experiments have been performed on V-M and SD-M, with the condition of a constant-temperature 60 °C and relative humidity of 90%, shown in Fig. S10. Results shown the thermochromism nearly vanishes after about 15 days’ treatment for V-M and 36 days for SD-M, pronounced the improvement in durability with spinodal decomposition. For a comparison, protective Al_2_O_3_ coating can be thermochromic only for 7 days^43^, while WO_3_ coatings are durable even after 20 days^44^.

## Conclusions

Fabrication of self-assembled lamella multilayer VO_2_-TiO_2_ thin films was realized by a two-steps approach of room temperature magnetron sputtering - post annealing *via* spinodal decomposition mechanism. The spinodally decomposed TVO film has been achieved on different sapphire substrates (A-plane (11–20) sapphire, R-plane (1–102) sapphire, C-plane (0001) sapphire, M-plane (10-10) sapphire substrates). All decomposed films display nano-scaled multilayer structures with well-ordered alternating Ti-rich and V-rich parallel layers, while the multilayered TVO structure and substrate have different orientations. The decomposed films tend to be slanted on A- and R-sapphire, perpendicular on C-sapphire, and parallel on M-sapphire. The spinodal decomposition is governed by a diffusional mechanism.

The annealing at 500 °C for 10 h can lead to a complete phase separation via our RTS-A method. All spinodally decomposed TVO films display thermochromic properties, and hence, our new technique has a high potential to be applied on smart coatings. The decomposed films on A-, R-, and M-sapphire also show both the reduced transition temperature and the narrowed hysteresis loop of the thermochromic V-rich layer. The spinodally decomposed TVO thin film bears anisotropic stress, and thus, it is promising to be utilized in optical switching. Moreover, the spinodally decomposed films display significantly better durability than VO_2_ thin film. These self-assembled multilayer-structures and outstanding regulating optical properties indicate that spinodal decomposition is an ideal approach for fabricating VO_2_-based smart windows.

## Methods

Amorphous TVO thin films (A-A, A-R, A-C, A-M) were first deposited by magnetron sputtering (ULVAC, ACS-4000-C4) on A-sapphire, R-sapphire, C-sapphire, M-sapphire substrates respectively, and then annealed for spinodal decomposition. The deposition of these amorphous TVO samples was carried out by co-sputtering from a VO_2_ ceramic target at 70 W dc power and a TiO_2_ ceramic target at 100 W rf power at room temperature with Ar and O_2_ flow of 39 and 1 sccm, respectively. Representative spinodal decomposition (SD) samples (SD-A, SD-R, SD-C, SD-M) were then obtained by annealing the amorphous samples at 500 °C for 10 h at 1 mTorr. For comparison, crystalline solid solution TVO (C-A, C-R, C-C, C-M) were prepared under nearly identical conditions as the amorphous samples, except that the substrate temperature was kept at 450 °C while sputtering. Single component VO_2_ thin film samples (V-A, V-R, V-C, V-M) and TiO_2_ thin film samples (T-A, T-R, T-C, T-M) were prepared at 450 °C by alternatively DC sputtering a VO_2_ ceramic target at 70 W and RF sputtering a TiO_2_ ceramic target at 100 W. A schematic diagram of the preparation processes can be found in Fig. S1(a).

X-ray photoemission spectroscopy (XPS) analysis was conducted on ThermoFisher ESCAlab250 to detect the elementary composition and content of TVO. Thin film X-ray diffraction (XRD) analysis was carried out on a Rigaku Ultima IV diffractometer with Cu Kα radiation (*λ* = 1.5418 Å) using the *θ*-2*θ* scanning model. Transmission electron microscopy (TEM) observations were carried out with the electron microscope (FEI, TECNAI G^2^ F20) equipped with an EDS analyzer (OXFORD, X-Max^N^). The optical transmittance of the films in the wavelength range from 350 nm to 2600 nm at 20 °C and 90 °C was measured using a UV-Vis spectrophotometer (HITACHI, UV-4100). The temperature was measured precisely with a temperature sensor in contact with the surface of films and controlled by a temperature controlling unit.

In addition, summary of the acronyms in this manuscript has been listed at the last of the supporting information in Table S3.

### Data availability statement

All data generated or analysed during this study are included in this published article and its Supplementary Information files.

## Electronic supplementary material

supporting information
