# Supplementary material for: Multi-nanolayered VO2/Sapphire Thin Film via Spinodal Decomposition
Source: Sci Rep. 2018 Mar 28;8:5342. doi: 10.1038/s41598-018-23412-4 (PMC5871865; doi:10.1038/s41598-018-23412-4)
Supplement: Supplementary file 1 — supporting information [file 41598_2018_23412_MOESM1_ESM.doc]

Supplementary Material

**Multi-nanolayered VO_2_/Sapphire Thin Film via Spinodal Decomposition**

Guangyao Sun,^a,b†^ Xun Cao,^a,†*^ Yuanzheng Yue,^c,d^ Xiang Gao,^e^ Shiwei Long,^a,b^ Ning Li, ^a,f^ Rong Li,^a^ Hongjie Luo,^g^ and Ping Jin^a,h*^

^a^ State Key Laboratory of High Performance Ceramics and Superﬁne Microstructure, Shanghai institute of Ceramics, Chinese Academy of Sciences, Dingxi 1295, Changning, Shanghai, 200050, China.

^b^ University of Chinese Academy of Sciences, Beijing, 100049, China.

^c^ Section of Chemistry, Aalborg University, DK-9220 Aalborg, Denmark.

^d^ State Key Laboratory of Silicate Materials for Architectures, Wuhan University of Technology, Wuhan 430070, China.

^e^ Thin Films and Nanostructures Group, Materials Science and Technology Division, Oak Ridge National Laboratory, 1 Bethel Valley Rd., Oak Ridge, Tennessee 37831, USA

^f^ Department of Materials Science and Engineering, College of science , China University of Petroleum Beijing, No. 18 Fuxue RD, Beijing 102249, China

^g^ School of Materials Science and Engineering, Shanghai University, 99 Shangda Road, Shanghai 200444, China

^h^ Materials Research Institute for Sustainable Development, National Institute of Advanced Industrial Science and Technology, Nagoya 463-8560, Japan

1. **Schematic diagram of the synthetic routes.**

Our experiments show that both room temperature sputtering-annealing (RTS-A) and quick quenching-annealing (Q-A) methods can induce spinodal decomposition in TVO system (**Fig. S1**). Since the Q-A method make both the substrates and the thin films easily to fracture upon quenching, we prefer the RTS-A method in magnetron sputtered thin films. Meanwhile, the synthesis process can be simplified.


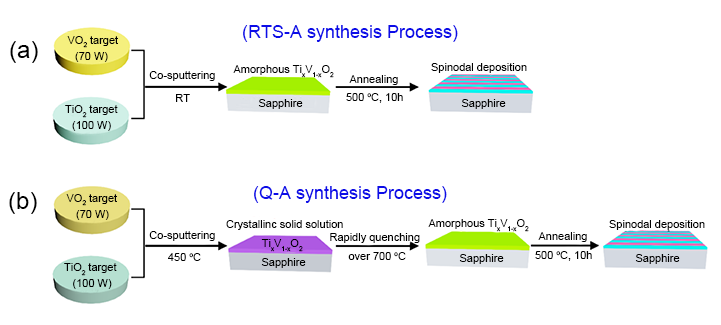


**Fig. S1** Schematic diagram of the synthetic route of (a) room temperature sputtering-annealing (RTS-A) synthesis process, (b) quick quenching-annealing method (Q-A).

1. **XPS analyses of amorphous samples**

Thin film composition was determined by XPS analysis conducted on the amorphous samples (A-A, A-R, A-C, A-M) and results are shown in supplementary **Fig. S2**. The spectra were calibrated by the C 1s peak (284.6 eV) considering environmental hydrocarbon contamination on the sample surface, thus there is one C 1s peak in the survey spectrum of **Fig. S2(a)**. Except the C 1s peak, there are no impurity peaks indicating the high purity of all samples. In **Fig. S2(b and c)**, all peaks of Ti 2p^3/2^ are centered at 458.7 eV, and all V 2p^3/2^ are at 516.1 eV, agreeing well with the standard positions for Ti^4+^ in TiO_2_ (458.80 eV) and V^4+^ in VO_2_ (516.30 eV). This confirms the tetravalency of both Ti and V, i.e., the Ti*_x_*V_1-_*_x_*O_2_ formation. In addition, the peak areas corresponding to V 2p and Ti 2p in **Fig. S2(b and c)** were employed to estimate the value of *x*. These results give a molar ratio of Ti to V of 0.33 for all the samples, therefore the composition can be written as Ti_0.25_V_0.75_O_2_.

**
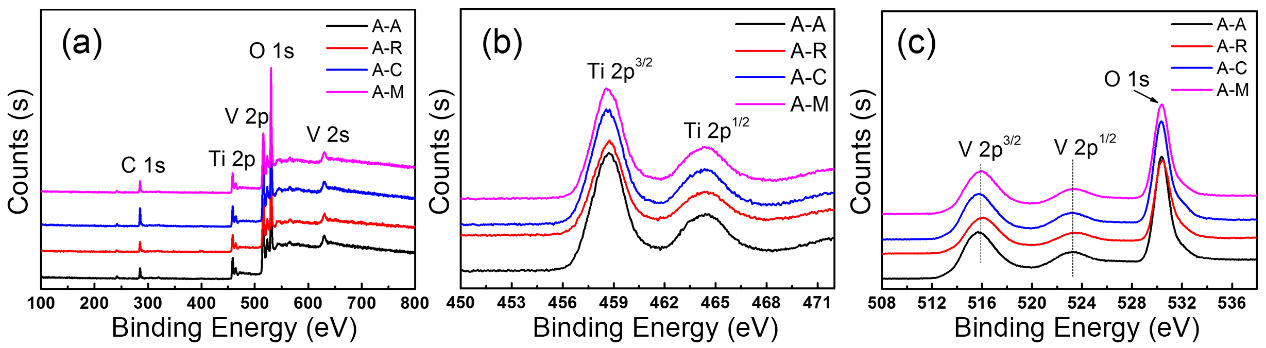
**

**Fig. S2** (a) Survey XPS spectra of amorphous samples (A-A, A-R, A-C, A-M) and high-resolution scan of (b) V 2p and O 1s, (c) Ti 2p spectra.

1. **XRD patterns of all the samples**

XRD results of all crystalline samples are shown in **Fig. S3** and are classified into four groups in terms of the sapphire type. **Fig. S3** confirms the orientation as only the peaks expected for each sample are observed, e.g., (101) peak for both A-sapphire samples (V-A, T-A, C-A) and R-sapphire samples (V-R, T-R, C-R), (200) peak for C-sapphire samples (V-C, T-C, C-C), and (002) peak for M-sapphire samples (V-M, T-M, C-M). All the peaks observed in the solid solution samples are located between the VO_2_ and TiO_2_, indicating the formation of crystalline solid solution (C-A, C-R, C-C, C-M) with tetragonal structure. The spinodally decomposed samples can be regarded as the crystalline solid solutions, exhibiting the solid solution peaks that split into two sets after the composition modulation. The peaks at the lower and the higher angles are indexed to Ti-rich and V-rich phases, respectively. Both the Ti-rich and V-rich peaks are located between the peak of pure VO_2_ and that of TiO_2_, confirming that the composition of the separated phases are V-doped TiO_2_ and Ti-doped VO_2_, respectively. The SD-C sample does not exhibit separated peaks because the separated peaks of SD-C are too close to be distinguished and its decomposition structure is identified by EDS measurements (**Fig. 2**).

**
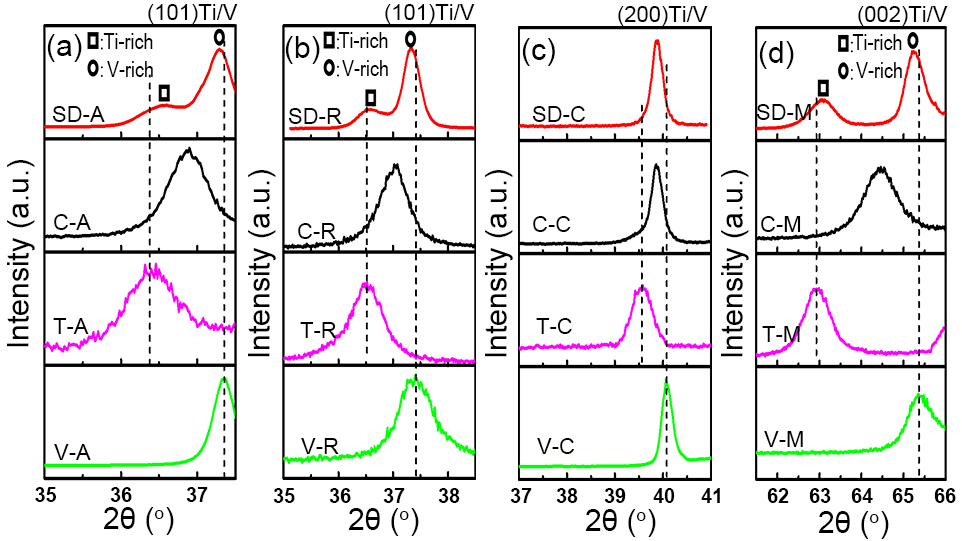
**

**Fig. S3** XRD patterns of samples. Single component VO_2_ thin film samples (V-A, V-R, V-C, V-M) are shown in green lines, single TiO_2_ samples (T-A, T-R, T-C, T-M) in purple lines, crystalline solid solution TVO (C-A, C-R, C-C, C-M) in black and spinodally decomposed samples in red. Amorphous samples (A-A, A-R, A-C, A-M) are not shown for there is no diffraction peak can be found.

1. **Cross-sectional STEM images for spinodally decomposed films**

**
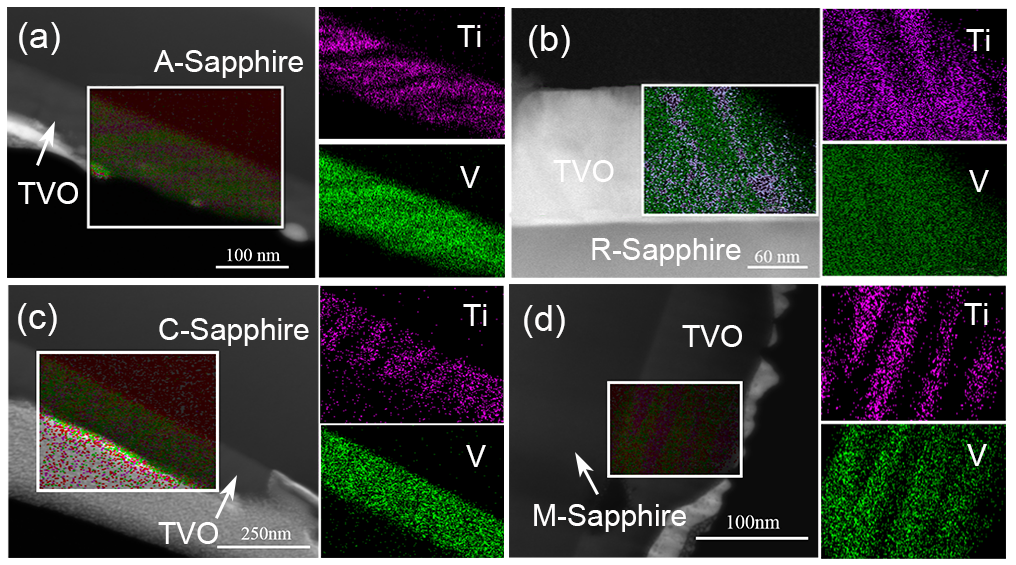
**

**Fig. S4** STEM images for spinodally decomposed samples of (a) SD-A (b) SD-R (c) SD-C and (d) SD-M, and the EDS elemental mapping results of Ti and V for the selected area (white square).

1. **High resolution TEM images of SD-M sample**

**Fig. S5** shows the high-resolution TEM (HRTEM) analyses on SD-M. The overall thickness of the TVO film was measured to be about 120 nm. In **Fig. S5(b)**, an interface formed between the Ti-rich and V-rich layers is evident, as indicated by a dashed line. The two layers are determined by a combined measurement of the out-of-plane lattice spacings, which are 2.95 Å and 2.87 Å, and correspond to the (001) planes of the Ti-rich and V-rich phases, respectively. According to the XRD patterns of SD-M (**Fig. S3(d)**), the (002) diffraction peak lies at 63.08 ^o^ (*d*= 1.47 Å) for the Ti-rich phase and 65.22 ^o^ (*d*= 1.43 Å) for the V-rich phase. The measured lattice spacings in **Fig. S5(b)** match well with those determined by XRD. The crystal orientation between the two phases is also revealed by performing the fast Fourier transform (FFT) analysis of the selected area (**Fig. S5(c, d)**). These results indicate that the spinodal phase separation, giving rise to an alternating Ti-rich and V-rich multilayer structure, has indeed taken place during the annealing process of amorphous TVO thin films.

**
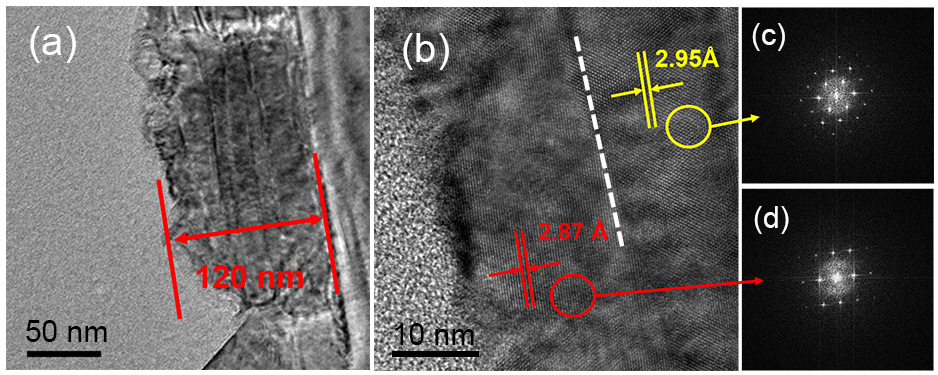
**

**Fig. S5** (a, b) High resolution TEM images of SD-M sample, (c, d) FFT of the selected grains in image (b).

1. **Cross-sectional STEM images for SD-M samples with different annealing time**


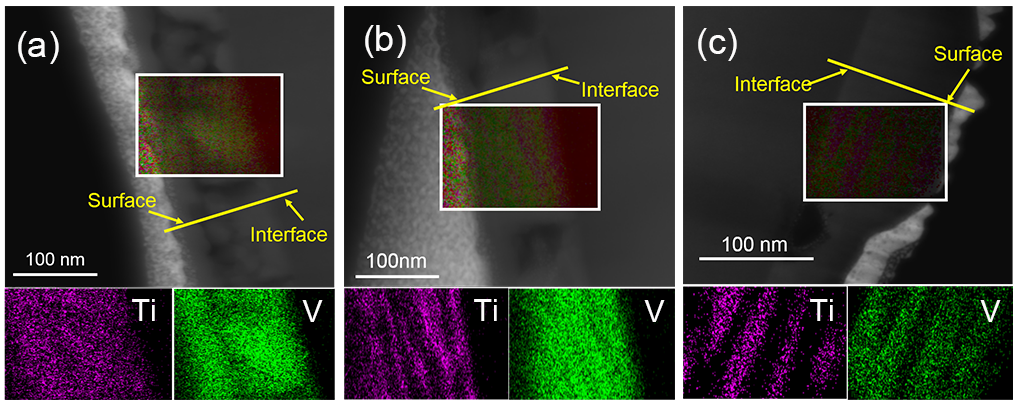


**Fig. S6** STEM images for spinodally decomposed sample SD-M with annealing time of (a) 1h (b) 5h and (c) 10h. The EDS elemental mapping of Ti and V was conducted on the selected area (white square) and line scanning of Ti and V on the selected line (yellow line).

1. **XRD evolution of SD-M samples with different annealing time**

XRD tests of SD-M samples with different annealing time are taken under the same condition, thus the peak intensity can reveal the relative content of both Ti-rich and V-rich phases between samples. XRD patterns are shown in **Fig. S7(a)** while variation tendency of [characteristic](javascript:void(0);) [peak](javascript:void(0);)s in **Fig. S7(b)**. Peak intensity increases when annealing time <10h and keeps almost the same afterwards. Together with optical spectra (**Fig. S8(b)**), we confirm that the sufficient annealing time is about 10h under 500 ^o^C, and the final separated phases are thermodynamically stable at the annealing temperature.


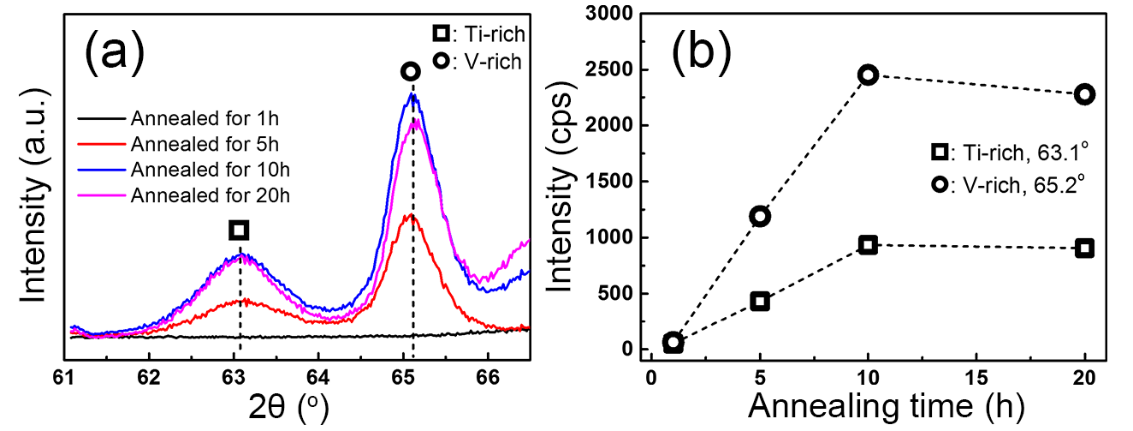


**Fig. S7** (a) XRD patterns of samples annealed for 1h, 5h, 10h and 20h. Dash lines show the decomposition peaks of Ti-rich (63.1 ^o^) and V-rich (65.2 ^o^) peaks. (b) Detailed investigation of XRD peaks evolution of Ti-rich and V-rich phases.

1. **Optical properties of both SD-M samples annealed for different time**

Optical properties of both the SD-M samples with different annealing time and the crystalline solid solution sample C-M are shown in **Fig. S8** and **Table S1**. C-M shows a limited gradual change of optical transmittance upon heating or cooling, i.e., no sharp transition as a function of temperature, and hence, no SMT performance, as reported elsewhere.^1^ The SMT performance of the C-M sample appears when it is annealed for >1 h, and it is further enhanced, and then saturated with extending the annealing time (see **Fig. S8(b and c)**). In **Fig. S8(a)**, a gradual red shift in spectra with increasing the annealing time (1, 5, and 10 h) is observed, indicating the gradual formation of a Ti-rich layer as discussed above. In **Table S1**, the gradual increase *ΔT_2000nm_* reveals the gradual formation of a V-rich layer, whereas the gradual decrease in transition temperature (*T_c-heating_*) reveals the stress decrease during the annealing progress. The 5h sample has an extremely large hysteresis loop width due to incomplete phase separation, since the coherent solid solution hinders the structure transformation during SMT. All these results support the diffusional mechanism of spinodal decomposition (**Fig. 3(a)**).


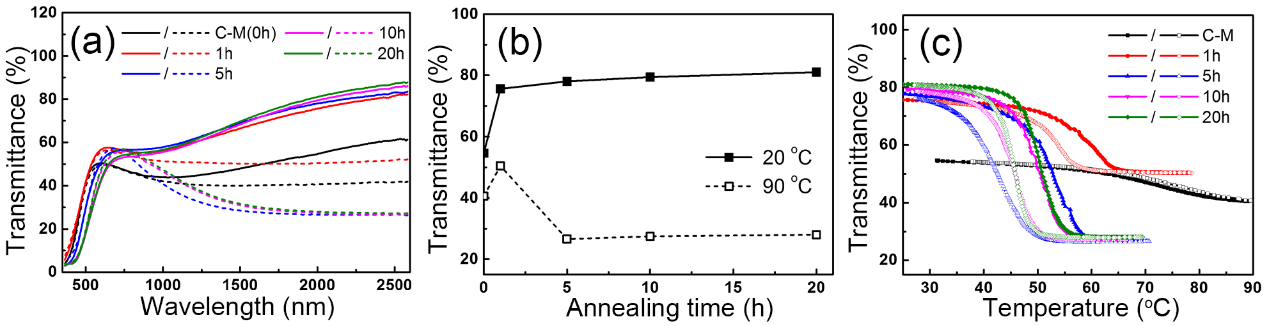


**Fig. S8** (a) Optical transmittance spectra at 20 ^o^C and 90 ^o^C of SD-M samples with different annealing time (0h, 1h, 5h, 10h, 20h). (b) Transmittance evolution of samples at 2000nm. (c) Transmittance hysteresis loops of samples at 2000nm.

Table S1. Optical properties of both SD-M samples annealed for different time

| Sample | *T_lum_*(%) | |  | *T_sol_*(%) | | *ΔT_sol_*  (%) | *ΔT_2000nm_*  (%) | *T_c-heating_*  (^o^C) | *T_c-cooling_* | *ΔT_c_* |
| --- | --- | --- | --- | --- | --- | --- | --- | --- | --- | --- |
|  | 20^o^C | 90^o^C |  | 20^o^C | 90^o^C |  |  |  | (^o^C) | (^o^C) |
| C-M | 47.6 | 46.3 |  | 43.3 | 40.9 | 2.4 | 14.0 | --- | --- | --- |
| 1h | 51.3 | 50.4 |  | 52.2 | 47.9 | 4.3 | 25.1 | 59.8 | 54.3 | 5.5 |
| 5h | 42.8 | 44.1 |  | 49.3 | 39.5 | 9.8 | 51.4 | 53.0 | 42.5 | 10.5 |
| 10h | 32.3 | 35.9 |  | 44.7 | 38.1 | 6.6 | 51.9 | 50.1 | 46.3 | 3.8 |
| 20h | 32.9 | 36.6 |  | 45.5 | 38.8 | 6.7 | 52.8 | 50.3 | 46.1 | 4.2 |

1. **Estimated optical energy band gap**

Spinodal decomposition changes the optical band gap of the TVO films. The estimated optical energy band gap (Eg) is determined from optical transmittance spectra (**Fig. 4(a~d)**) with the following formula:

(αhυ)^2^ = A(hυ-Eg)

Where hυ = 1240/λ, α(λ) is the optical absorption coefficient and is evaluated using Beer - Lambert’s function ^2^:

α(λ) = -ln[T/(1-R)]

Thus Eg value can be obtained by using the linear extrapolation of (αhυ)^2^ vs hυ and exploring the intercept when α = 0. Results are shown in **Fig. S9**.

In **Fig. S9**, the band gaps before and after annealing are shown, and the single layer VO_2_ samples are also shown for comparison. All amorphous TVO samples (before annealing) possess the same large band gap of 2.87eV and the gap narrows after annealing to 2.15eV for SD-A, 1.85eV for SD-R, 2.39eV for SD-C and 2.20eV for SD-M. The band gap changes during the amorphous-to-crystalline transition reveals the optical absorption among the transition, convincing the efficacy of spinodal decomposition in solar modulation ability enhancement. All single layer VO_2_ samples show the same band gap of 2.27eV, compared spinodal decomposition samples with them, the SD-A, SD-R and SD-M samples appear to be red shift while SD-C shows blue shift. The data are in accordance with the transmittance spectra.


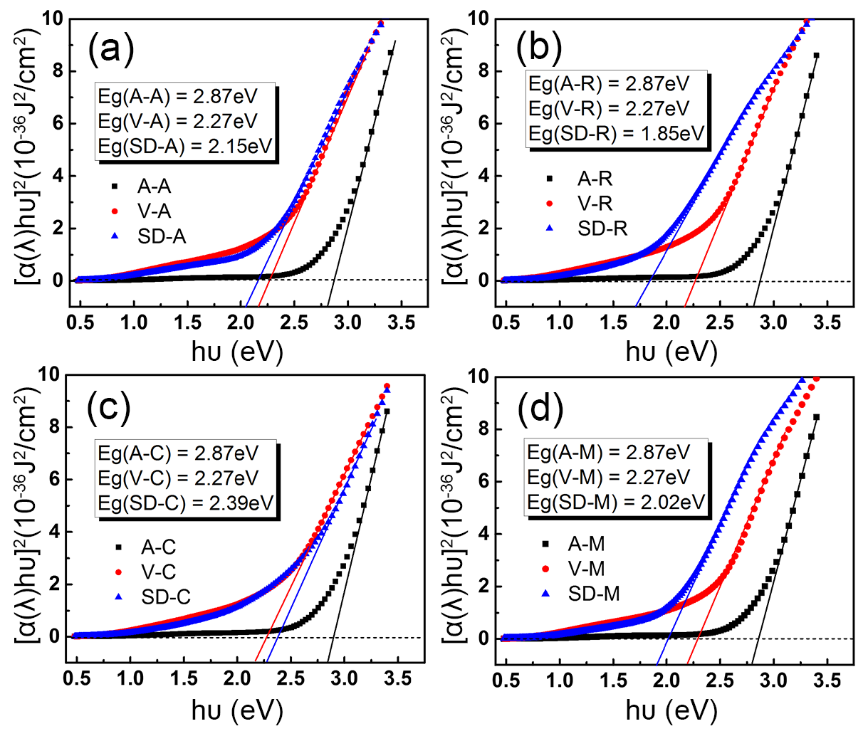


**Fig. S9** Plot of the (αhυ)^2^ vs hυ relationship. Amorphous TVO samples (A-A, A-R, A-C, A-M) are in black squares, single layer VO_2_ samples (V-A, V-R, V-C, V-M) are in red circles and spinodal decomposition samples (SD-A, SD-R, SD-C, SD-M) are in blue triangles. The estimated optical energy band gap are shown.

1. **Recently reported VO_2_-based thermochromic films prepared by magnetron sputtering**

Table S2. Optical properties of recently reported VO_2_-based thermochromic films prepared by magnetron sputtering.

| Sample | *T_lum_*(%) | |  | *T_sol_*(%) | | *ΔT_sol_*  (%) |
| --- | --- | --- | --- | --- | --- | --- |
|  | 20^o^C | 90^o^C |  | 20^o^C | 90^o^C |  |
| TiO_2_/VO_2_/TiO_2_^3^ | 30.10 | 27.80 |  | 33.80 | 23.60 | 10.20 |
| TiO_2_/VO_2_^3^ | 32.40 | 31.30 |  | 37.20 | 28.60 | 8.60 |
| VO_2_/V_2_O_3_^4^ | 38.30 | 39.40 |  | 42.10 | 38.10 | 4.00 |
| VO_2_/WO_3_^5^ | 43.60 | 42.20 |  | 42.90 | 37.50 | 5.40 |
| VO_2_/SiO_2_^6^ | 27.60 | 26.30 |  | 25.10 | 18.80 | 6.30 |
| VO_2_/SnO_2_^6^ | 30.80 | 29.40 |  | 28.80 | 22.80 | 6.00 |
| VO_2_^7^ | 34.60 | - |  | - | - | 9.80 |

1. **Durability experiments for spinodally decomposed films**

To evaluate the stability of spinodally decomposed films, durability experiments have been performed. V-M (**Fig. S10(a, b)**) and SD-M (**Fig. S10(c, d)**) were placed in a constant-temperature humidity chamber at 60 ^o^C and relative humidity of 90% to accelerate the corrosion process, and optical transmittance measurements were carried out once a day.

Typical spectra are measured during the deterioration of V-M (**Fig. S10(a))**. The transmittance of the V-M sample at both the wavelength of 550 nm and 2000 nm with ageing time were measured (**Fig. S10(b))**. The deterioration of the single VO_2_ thin film proceeds quickly, and its transmittance in both the visible and infrared regions increases gradually with ageing time. The transmittance difference at 2000 nm between the lower temperature (20 °C) and the higher temperature (90 °C) phases decreases with ageing time. Finally, the thermochromism nearly vanishes after about 15 days treatment in the chamber. For SD-M, as shown in **Fig. S10(c, d)**, the spectra remain nearly unchanged during the first 10 days and afterwards change drastically, indicating the deterioration of film. The complete deterioration appears after 36 days, and is far more pronounced than V-M.

**
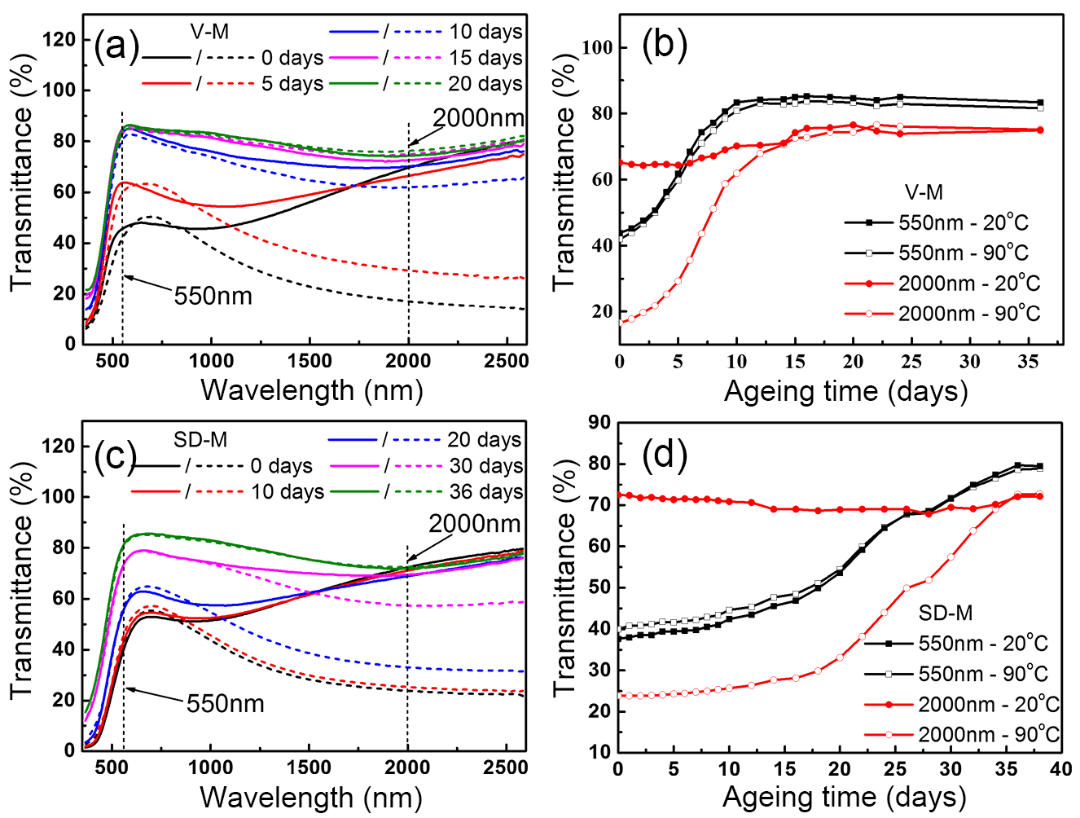
**

**Fig. S10** (a, c) Transmittance spectra at 20 ^o^C and 90 ^o^C of (a) V-M and (c) SD-M after being placed in ageing conditions of 60 ^o^C and relative humidity of 90% for several days. (b, d) Evolution of transmittance at 550nm and 2000nm after ageing.

1. **Others:**

Table S3. Summary of the acronyms in this manuscript

| Category | Acronyms | Full name |
| --- | --- | --- |
| Sample mane | TVO | TiO_2_/VO_2_ thin film system |
|  | A-A | Amorphous TVO on A-plane (11-20) sapphire |
|  | A-R | Amorphous TVO on R-plane (1-102) sapphire |
|  | A-C | Amorphous TVO on C-plane (0001) sapphire |
|  | A-M | Amorphous TVO on M-plane (10-10) sapphire sapphire |
|  | C-A | Crystalline solid solution TVO on A-plane sapphire |
|  | C-R | Crystalline solid solution TVO on R-plane sapphire |
|  | C-C | Crystalline solid solution TVO on C-plane sapphire |
|  | C-M | Crystalline solid solution TVO on M-plane sapphire |
|  | V-A | VO_2_ thin film on A-plane sapphire |
|  | V-R | VO_2_ thin film on R-plane sapphire |
|  | V-C | VO_2_ thin film on C-plane sapphire |
|  | V-M | VO_2_ thin film on M-plane sapphire |
|  | T-A | TiO_2_ thin film on A-plane sapphire |
|  | T-R | TiO_2_ thin film on R-plane sapphire |
|  | T-C | TiO_2_ thin film on C-plane sapphire |
|  | T-M | TiO_2_ thin film on M-plane sapphire |
|  | SD-A | Spinodal decomposition samples on A-plane sapphire |
|  | SD-R | Spinodal decomposition samples on R-plane sapphire |
|  | SD-C | Spinodal decomposition samples on C-plane sapphire |
|  | SD-M | Spinodal decomposition samples on M-plane sapphire |
| [Synthetic](javascript:void(0);) [route](javascript:void(0);) | RTS-A | Room temperature sputtering - annealing method |
|  | Q-A | Quenching – annealing method |
| Optical properties | *T_lum_* | Luminous transmittance |
|  | *T_sol_* | Solar transmittance |
|  | *ΔT_sol_* | Solar modulating ability |
|  | *ΔT_2000nm_* | Transmittance difference at 2000nm |
|  | *T_c_* | Phase-transition [temperature](javascript:void(0);) |
|  | *T_c-heating_* | [Phase-transition](javascript:void(0);) [temperature](javascript:void(0);)s of heating lines |
|  | *T_c-cooling_* | [Phase-transition](javascript:void(0);) [temperature](javascript:void(0);)s of cooling lines |
|  | *ΔT_c_* | The hysteresis loop width, *ΔT_c_* = *T_c-heating_* - *T_c-cooling_* |
| Material characterization methods | XRD | X-ray diffraction |
|  | TEM | Transmission electron microscopy |
|  | STEM | Scanning transmission electron microscopy |
|  | EDS | Energy Disperse Spectroscopy |
|  | XPS | X-ray photoemission spectroscopy |

**Reference**

(1) Li, J.; Dho, J., Controlling metal–insulator transition in the hetero-epitaxial VO_2_/TiO_2_ bilayer grown on Al_2_O_3_. *J. Cryst. Growth* **2010,** *312* (22), 3287-3291.

(2) Li, Y.; Ji, S.; Gao, Y.; Luo, H.; Jin, P., Modification of Mott phase transition characteristics in VO_2_@TiO_2_ core/shell nanostructures by misfit-strained heteroepitaxy. *ACS Appl. Mater. Interfaces* **2013,** *5* (14), 6603-6614.

(3) Zheng, J.; Bao, S.; Jin, P., TiO_2_(R)/VO_2_(M)/TiO_2_(A) multilayer film as smart window: Combination of energy-saving, antifogging and self-cleaning functions. *Nano Energy* **2015,** *11*, 136-145.

(4) Sun, G. Y.; Cao, X.; Gao, X.; Long, S. W.; Liang, M. S.; Jin, P., Structure and enhanced thermochromic performance of low-temperature fabricated VO_2_/V_2_O_3_ thin film. *Appl. Phys. Lett.* **2016,** *109* (14), 143903.

(5) Long, S.; Zhou, H.; Bao, S.; Xin, Y.; Cao, X.; Jin, P., Thermochromic multilayer films of WO_3_/VO_2_/WO_3_ sandwich structure with enhanced luminous transmittance and durability. *RSC Adv.* **2016,** *6* (108), 106435-106442.

(6) Zhu, B.; Tao, H.; Zhao, X., Effect of buffer layer on thermochromic performances of VO_2_ films fabricated by magnetron sputtering. *Infrared Phys. Technol.* **2016,** *75*, 22-25.

(7) Choi, Y.; Jung, Y.; Kim, H., Low-temperature deposition of thermochromic VO_2_ thin films on glass substrates. *Thin Solid Films* **2016,** *615*, 437-445.
